# Cup-to-Disc Ratio Is Associated with Disability in Multiple Sclerosis: A Combined OCT and Subjective Visual Vertical Study

**DOI:** 10.3390/medicina62061158

**Published:** 2026-06-14

**Authors:** Ieva Vienažindytė, Tautvydas Klėgėris, Ingrida Ulozienė, Diego Kaski, Brigita Glebauskienė, Renata Balnytė

**Affiliations:** 1 Department of Neurology, Medical Academy, Lithuanian University of Health Sciences, 44307 Kaunas, Lithuania; renata.balnyte@lsmu.lt; 2 Department of Otorhinolaryngology, Faculty of Medicine, Lithuanian University of Health Sciences, 44307 Kaunas, Lithuania; tautvydas.klegeris@lsmu.lt (T.K.); ingrida.uloziene@lsmu.lt (I.U.); 3 Department of Clinical and Movement Neuroscience, University College London, London WC1E 6BT, UK; d.kaski@ucl.ac.uk; 4 Department of Ophthalmology, Medical Academy, Lithuanian University of Health Sciences, 44307 Kaunas, Lithuania; brigita.glebauskiene@lsmu.lt

**Keywords:** multiple sclerosis, optical coherence tomography, cup-to-disc ratio, retinal nerve fiber layer, neurodegeneration, disability

## Abstract

*Background and Objectives*: Non-invasive biomarkers reflecting neurodegeneration are increasingly important in multiple sclerosis (MS). Optical coherence tomography (OCT) provides quantitative measures of retinal structure, most commonly peripapillary retinal nerve fiber layer (pRNFL) thickness. However, the potential clinical relevance of optic nerve head morphology, including cup-to-disc ratio (CDR), remains insufficiently explored. We investigated associations between OCT-derived parameters, subjective visual vertical (SVV), and disability in MS. *Materials and Methods*: In this retrospective study, 100 patients with MS were included. OCT parameters (pRNFL thickness and area-based CDR) were analyzed at baseline and follow-up. Clinical disability was assessed using the Expanded Disability Status Scale (EDSS). Detailed optic neuritis history was not consistently available in the retrospective clinical records and therefore could not be systematically accounted for in the analyses. SVV was evaluated in 37 patients using a virtual reality–based protocol. Associations were assessed using Spearman correlation and linear regression analyses. Multivariable regression models were adjusted for age, sex, and follow-up duration. *Results*: pRNFL thickness was not associated with baseline EDSS (rho = −0.06, *p* = 0.55) or annualized EDSS change. Baseline CDR correlated with both baseline EDSS (rho = 0.30, *p* = 0.0065) and follow-up EDSS (rho = 0.46, *p* < 0.0001). In univariable regression analysis, baseline CDR was associated with follow-up EDSS (B = 3.33, R^2^ = 0.23, *p* < 0.0001), remaining significant after adjustment for age, sex, and follow-up duration (B = 2.59, 95% CI 1.26–3.92, *p* = 0.0002). No significant associations were observed between OCT parameters and SVV measures. *Conclusions*: Higher CDR values, but not pRNFL thickness, were associated with disability measures in this exploratory MS cohort. However, these findings should be interpreted cautiously because optic neuritis history could not be systematically accounted for and physiological optic disc variability may substantially influence CDR measurements.

## 1. Introduction

Multiple sclerosis (MS) is a chronic immune-mediated disease characterized by demyelination and neuroaxonal loss, leading to progressive disability [1,2]. Clinical assessment and MRI remain central to monitoring, but clinical evaluation is partly subjective, and repeated MRI is costly or sometimes contraindicated [1,2]. Therefore, non-invasive biomarkers reflecting neurodegeneration are of increasing importance [3].

Optical coherence tomography (OCT) allows high-resolution, in vivo quantification of retinal layers, particularly the peripapillary retinal nerve fiber layer (pRNFL) and macular ganglion cell–inner plexiform layer (GCIPL), which serve as structural proxies for axonal and neuronal integrity [4,5]. Retinal thinning occurs in eyes with optic neuritis (ON) and in clinically unaffected eyes, reflecting diffuse neurodegeneration in MS [4,5,6]. The retinal nerve fiber layer represents axons that converge at the optic nerve head (optic disc), where structural OCT measurements such as neuro-retinal rim area and disc area can provide supplementary information on optic nerve integrity; in MS patients, neuro-retinal rim area is decreased and correlates with RNFL thinning and visual function [7].

Retinal thinning in MS is thought to reflect neuroaxonal degeneration occurring both locally and along the visual pathway through trans-synaptic mechanisms [8,9,10].

Optic neuritis is a common MS manifestation, often representing the initial demyelinating event, leading to acute visual loss and permanent axonal damage in some patients [11]. OCT studies demonstrate significant thinning of pRNFL and GCIPL following ON, with longitudinal studies showing progressive thinning even in non-ON eyes [12].

OCT-derived retinal measures correlate with clinical disability and brain atrophy. Reduced pRNFL thickness is associated with higher EDSS scores, and accelerated GCIPL thinning predicts disability progression [13,14]. Longitudinal studies confirm that retinal thinning mirrors global brain atrophy [15]. Standardization of OCT protocols and normative datasets improve measurement reproducibility and clinical applicability [16]. Integration of OCT metrics into multidimensional platforms such as MS BioScreen may facilitate individualized prognosis and therapeutic decision-making [2].

Beyond retinal layer thickness, optic nerve head morphology may provide complementary information regarding structural changes occurring at the level of the optic nerve head in MS. Previous studies have reported abnormal cup-to-disc ratio (CDR) values in MS patients, suggesting that optic nerve head changes may accompany neuroaxonal degeneration [7]. However, the relationship between CDR and neurological disability remains insufficiently explored.

Balance dysfunction and impaired spatial orientation are common in MS and may partly reflect altered perception of gravitational verticality [17,18,19,20,21,22,23].

In the present study, we investigated associations between retinal structural changes, specifically pRNFL thickness and cup-to-disc ratio, SVV (static and dynamic) measures, and disability in MS. In addition, we aimed to evaluate the relationship between OCT-derived parameters and clinical disability, and to determine whether optic nerve head morphology provides additional clinically relevant information beyond conventional retinal thickness measurements.

While previous work has primarily focused on pRNFL thickness and MRI-derived measures in MS, the present study specifically evaluates optic nerve head morphology, particularly area-based CDR, together with SVV as a functional measure of spatial orientation.

## 2. Materials and Methods

### 2.1. Study Design and Population

Patients with a diagnosis of multiple sclerosis (MS) according to the 2010 or 2017 revised McDonald criteria, treated at the Neurology Department and Outpatient Clinic of the Lithuanian University of Health Sciences Hospital Kauno Klinikos (LUHS Hospital KK), were eligible for inclusion. The study was conducted in accordance with the Declaration of Helsinki and the Law of the Republic of Lithuania on Ethics of Biomedical Research and was approved by the Kaunas Regional Bioethics Committee (protocol code P1-BE-2-113/2024, date of approval 24 July 2025). This was a retrospective observational study based on routinely collected clinical and imaging data.

This retrospective study analysed medical documentation from the Multiple Sclerosis Centre of the Neurology Clinic of the Lithuanian University of Health Sciences, including electronic hospital records and imaging data archives (MedDream system (Softneta, Vilnius, Lithuania)). Some participants may overlap with previously reported retrospective OCT cohorts from our centre; however, the present analysis addresses a distinct research question focused on optic nerve head morphology and SVV measures rather than pRNFL–MRI associations. Demographic (age, sex) and clinical data (disease course, disability assessed using the Expanded Disability Status Scale [EDSS]) were collected. Detailed information regarding previous optic neuritis episodes, including timing and affected eye, was not consistently available in the retrospective clinical records and therefore was not included in the primary analyses. Inclusion criteria were: (1) confirmed MS diagnosis according to the McDonald criteria, (2) availability of at least one OCT examination including pRNFL and optic nerve head measurements, and (3) availability of corresponding neurological assessment including EDSS evaluation.

Where available, data from two time points were included: the earliest available OCT examination, considered baseline for the purposes of this study, and the most recent follow-up OCT examination. Follow-up duration was defined as the interval between baseline OCT examination and the most recent available EDSS assessment. EDSS values were matched to OCT examinations using the temporally closest available neurological assessment. The median interval between OCT examination and corresponding EDSS assessment was 119 days (IQR 22–252) at baseline and 186 days (IQR 91–352) at follow-up.

A total of 128 patients treated between 2020 and 2025 were initially screened. Exclusion criteria included insufficient clinical documentation and ocular conditions potentially affecting OCT measurements. Twelve patients were excluded due to insufficient clinical documentation (e.g., missing EDSS scores or OCT data). An additional 16 patients were excluded due to ocular conditions that could significantly affect OCT measurements (e.g., ischemic optic neuropathy, diabetic retinopathy, retinal detachment, advanced cataract, vitreous haemorrhage).

A total of 100 patients met the inclusion criteria and were included in the final analysis.

### 2.2. OCT Acquisition and Parameters

Optic nerve head (ONH) and peripapillary retinal nerve fiber layer (pRNFL) measurements were obtained using Triton DRI OCT (Topcon Corporation, Tokyo, Japan). Recorded OCT parameters included average pRNFL thickness, optic disc area, rim area, and area-based cup-to-disc ratio (CDR). Only scans with adequate image quality and without major motion artifacts were included.

Area-based CDR was calculated as follows: CDR = (disc area − rim area)/disc area.

### 2.3. Data Preprocessing and Quality Control

To improve measurement consistency in this retrospective real-world dataset, predefined quality control criteria were applied to OCT measurements. Eyes with extreme pRNFL values (<50 µm or >120 µm) were flagged for manual review as potentially artifactual values substantially outside expected clinical ranges in MS cohorts [24]. Measurements considered inconsistent with accompanying OCT parameters or suggestive of segmentation artifacts were excluded according to established OCT quality control principles [25].

To reduce inter-eye statistical dependence, OCT parameters were analyzed at the patient level. For patient-level analyses, OCT parameters were summarized at each time point by averaging measurements from both eyes when bilateral data were available. Patient-level averaging was used to reduce inter-eye statistical dependence and avoid pseudo-replication in participant-level analyses. If only one eligible eye was available, that eye’s value was used. Inter-eye pRNFL asymmetry was additionally assessed as the absolute difference between eyes. Inter-eye pRNFL asymmetry ≥20 µm was explored in sensitivity analyses as a potential indicator of substantial unilateral optic nerve involvement.

**Figure A1:**
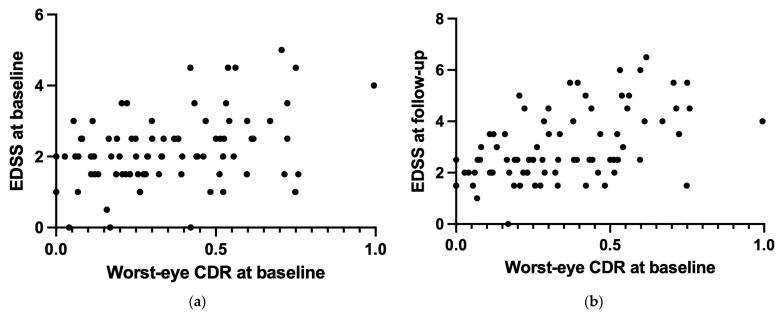
Scatterplots of worst-eye sensitivity analyses. (**a**) Association between worst-eye baseline CDR and baseline EDSS. (**b**) Association between worst-eye baseline CDR and follow-up EDSS.

Supplementary sensitivity analyses were additionally performed after quality control procedures using worst-eye OCT parameters, defined as the eligible eye with the lower pRNFL thickness and higher CDR value for each participant (Figure A1).

Additional consistency checks were performed for optic nerve head parameters. Cases where rim area equaled disc area (resulting in CDR = 0) were flagged and reviewed. These values were retained only if considered consistent with accompanying OCT parameters; otherwise, they were considered likely segmentation or reporting artifacts and excluded. Review decisions were based on consistency with pRNFL measurements, optic disc dimensions, and the overall plausibility of the optic nerve head profile. After quality control procedures, 5 measurements were excluded from OCT analyses.

OCT measurements were analyzed at the patient level using one summarized value per participant at each time point. Not all participants had complete longitudinal OCT data; therefore, sample sizes varied across analyses.

### 2.4. Subjective Visual Vertical Assessment as Clinical Marker

The SVV assessment protocol was adapted from our previous work [26], with modifications as described below.

SVV was assessed in a subgroup of 37 MS patients for whom virtual reality–based testing data were available. The subgroup did not differ significantly from the overall cohort in age, sex, or EDSS. Subjective visual vertical was assessed using the Oculus Quest 2 virtual reality headset, equipped with the SVV measurement application VIRVEST, which has been previously validated for both healthy individuals and patient populations [23,27]. Within the virtual environment, a three-dimensional rod was presented in the primary gaze position with an initial tilt randomly set between 10° and 15° from true gravitational vertical.

Participants used a wireless joystick to adjust the rod until it aligned with their perceived vertical and confirmed their response via a button press. Upon confirmation, the deviation angle was automatically recorded, and the subsequent trial was initiated. Each participant performed three testing conditions: static SVV, dynamic SVV with clockwise (CW) rotation, and dynamic SVV with counterclockwise (CCW) rotation. Each condition included six trials, resulting in a total of 18 SVV adjustments per participant.

In the static condition, the rod was displayed against a uniform black background without additional visual input. In contrast, during dynamic conditions, the rod appeared within a field of three-dimensional spheres distributed randomly throughout the visual scene. These spheres rotated in the frontal plane either clockwise or counterclockwise at a constant angular velocity of 10°/s, creating a moving visual context.

Quantitative data was obtained as the angular difference between true gravitational vertical and the participant’s adjusted rod position, measured automatically by the system with a precision of 0.1°. The mean SVV deviation angle was calculated from six trials for each test.

Visual dependence (VD), representing the influence of visual motion on verticality perception, was quantified as the average absolute difference between static and dynamic SVV measures [27,28,29,30]. Accordingly, VD was calculated using the formula:

VD = (|SVVcw − SVVstatic| + |SVVccw − SVVstatic|)/2.

### 2.5. Statistical Analysis

Statistical analyses were performed using GraphPad Prism version 11 (GraphPad Software, Boston, MA, USA). Normality of quantitative variables was assessed using the Shapiro–Wilk test. As most variables were not normally distributed (*p* < 0.05), non-parametric methods were applied where appropriate.

Continuous variables are presented as mean ± standard deviation or median with interquartile range (IQR), depending on data distribution.

Comparisons between independent groups were performed using the Mann–Whitney U test. Associations between variables were evaluated using Spearman correlation analysis.

Univariable and multivariable linear regression analyses were performed to assess associations between OCT parameters and disability measures. Multivariable linear regression models were constructed to adjust for potential confounders, including age, sex and follow-up duration. Covariates were selected a priori based on clinical relevance. Sensitivity analyses were performed after excluding participants with marked inter-eye pRNFL asymmetry (≥20 µm). Additional sensitivity analyses were performed after restricting analyses to EDSS–OCT pairs obtained within 180 days of each other.

Regression assumptions, including linearity, homoscedasticity, and residual distribution, were assessed using residual diagnostics. Residual distributions deviated from normality; therefore, regression findings were interpreted cautiously and primarily as exploratory effect-size estimates complementing the non-parametric correlation analyses. No imputation was performed due to the retrospective nature of the dataset and variable availability across follow-up visits. Given the exploratory and hypothesis-generating nature of the study, findings were interpreted primarily based on effect sizes, consistency across analyses, and biological plausibility rather than formal multiplicity-adjusted significance thresholds.

No formal a priori sample size calculation was performed because of the retrospective design. The final sample size was determined by the number of eligible patients with available clinical and OCT data. With 100 included participants, the study had approximately 85% power to detect a correlation coefficient of r = 0.30 at a two-sided α level of 0.05, indicating adequate power for moderate associations but limited power for small effects. The SVV subgroup analysis was exploratory and underpowered to detect small-to-moderate associations.

The primary analysis evaluated associations between baseline OCT parameters and follow-up EDSS.

## 3. Results

### 3.1. Study Population Characteristics and OCT Parameters

A total of 100 patients were included in the study. Due to missing longitudinal OCT data, the number of subjects varied across analyses. Baseline OCT measurements were available for all participants, whereas follow-up OCT data were available in a subset of patients (*n* = 51–57, depending on parameter). Because not all OCT parameters were available for all participants and complete longitudinal imaging data were not consistently available, sample sizes varied across analyses and are reported for each result individually.

The sample consisted of 31% males and 69% females. The mean age of the cohort was 34.6 ± 9.2 years. Among the included patients, 4 (4%) had primary progressive MS and 1 (1%) had secondary progressive MS, while the remaining patients had relapsing-remitting MS. One participant initially diagnosed with radiologically isolated syndrome fulfilled diagnostic criteria for relapsing-remitting MS during follow-up and was therefore included in the cohort. Median baseline EDSS was 2.0 [1.5–2.5], increasing to 2.5 [2.0–3.5] at follow-up. The median annualized EDSS change was 0.10 [0.00–0.40] over a median follow-up of 2.9 [1.2–6.4] years.

Mean baseline pRNFL thickness was 94.8 ± 14.1 µm, decreasing to 89.2 ± 15.5 µm at follow-up. Mean CDR increased slightly from 0.29 ± 0.19 at baseline to 0.31 ± 0.22 at follow-up (Table 1).

### 3.2. Association Between OCT Parameters and Disability

pRNFL thickness correlated weakly and inversely with baseline CDR (rho = −0.22, 95% CI −0.42 to −0.0001, *p* = 0.044, *n* = 83).

pRNFL thickness was not associated with disability measures including baseline EDSS (rho = −0.06, *p* = 0.55, n = 100) or with annualized EDSS change (rho = −0.07, *p* = 0.48, *n* = 100).

CDR correlated moderately with baseline EDSS (rho = 0.30, *p* = 0.0065, *n* = 83). Follow-up CDR was significantly associated with follow-up EDSS (rho = 0.49, *p* = 0.0003, *n* = 51).

Baseline CDR was also significantly associated with follow-up EDSS (rho = 0.46, *p* < 0.0001, *n* = 83). Sensitivity analyses restricted to EDSS–OCT intervals ≤ 180 days yielded comparable results. In this subset (*n* = 51), baseline CDR remained significantly associated with both baseline EDSS (rho = 0.44, 95% CI 0.18–0.64, *p* = 0.0013) and follow-up EDSS (rho = 0.50, 95% CI 0.26–0.69, *p* = 0.0002).

Supplementary worst-eye analyses yielded findings generally comparable to the primary patient-level averaged analyses. Worst-eye baseline CDR remained significantly associated with both baseline EDSS (rho = 0.32, *p* = 0.0038, *n* = 82) and follow-up EDSS (rho = 0.51, *p* < 0.0001, *n* = 82). In contrast, worst-eye baseline pRNFL showed no significant association with baseline EDSS and only a weak inverse association with follow-up EDSS (rho = −0.22, *p* = 0.031) (Table A1).

A weak, non-significant association was observed between baseline CDR and annualized EDSS change (rho = 0.21, *p* = 0.058, *n* = 83). Changes in CDR were not associated with changes in EDSS (rho = −0.07, *p* = 0.63, *n* = 43) or annualized EDSS progression (rho = −0.09, *p* = 0.57, *n* = 43).

**Figure 1:**
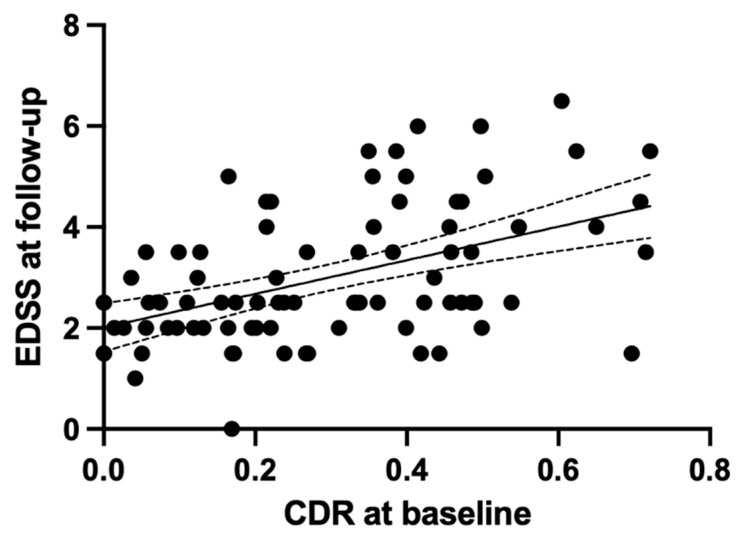
Association between baseline cup-to-disc ratio and follow-up EDSS. Each point represents one participant. The solid line represents the regression line, and dashed lines indicate 95% confidence intervals. EDSS—Expanded Disability Status Scale; CDR—cup-to-disc ratio.

In univariate regression analysis, baseline CDR was associated with follow-up EDSS (B = 3.33, R^2^ = 0.23, *p* < 0.0001). Each 0.1 increase in CDR corresponded to an approximate 0.33 increase in EDSS score (Figure 1).

**Figure 2:**
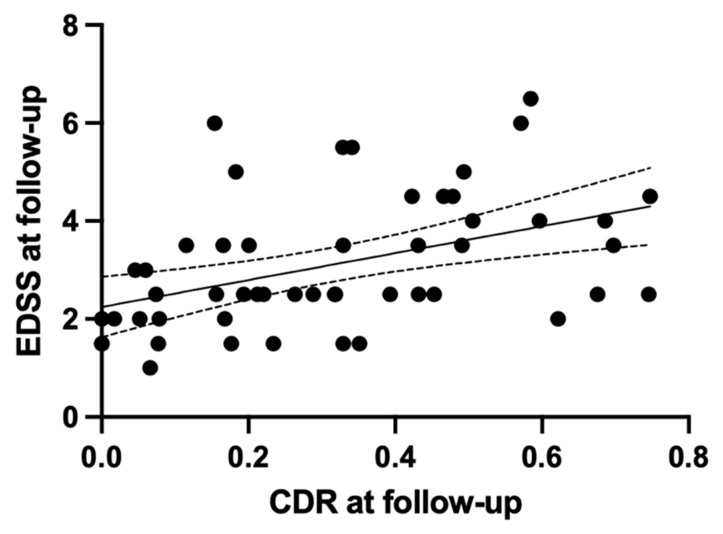
Association between follow-up cup-to-disc ratio and follow-up EDSS. Each point represents one participant. The solid line represents the regression line, and dashed lines indicate 95% confidence intervals. EDSS—Expanded Disability Status Scale; CDR—cup-to-disc ratio.

A similar association was observed for follow-up CDR (B = 2.75, R^2^ = 0.19, *p* = 0.0013) (Figure 2).

### 3.3. Multivariable Regression Analysis

In a multivariable linear regression model adjusted for sex, age and follow-up duration, baseline CDR remained independently associated with follow-up EDSS (B = 2.59, 95% CI 1.26–3.92, *p* = 0.0002).

Age was also independently associated with EDSS (B = 0.04, *p* = 0.005), whereas follow-up duration and sex were not significant predictors.

The overall model was significant (*p* < 0.0001) and explained 34% of the variance in EDSS (R^2^ = 0.34) (Table 2). Exclusion of participants with marked inter-eye asymmetry (>20 µm) did not materially alter the observed associations.

### 3.4. SVV Associations with pRNFL and CDR

During the static SVV test, the median absolute SVV deviations from gravitational vertical was 1.5° (0.1–17.9°). Significantly greater deviations were observed during both dynamic tests: 2.6° (0.6–18.0°) during the CW testing and 2.8° (0.4–30.3°) during the CCW testing (*p* < 0.001 for both conditions, Wilcoxon test). The median VD value in our patient sample was 1.87 (0.1–22.7). Participants with CDR values of ≥0.5 did not exhibit statistically significant larger SVV (in static and both dynamic conditions) errors nor VD scores (*p* > 0.05 for all test, Wilcoxon test). We did not identify any significant correlations between structural retinal changes and verticality perception measures (Table 3).

## 4. Discussion

In this exploratory study, optic nerve head morphology, as reflected by area-based cup-to-disc ratio (CDR), was associated with neurological disability in patients with MS, whereas pRNFL thickness was not.

Baseline CDR was associated not only with concurrent EDSS but also with EDSS at follow-up and remained associated after adjustment with follow-up EDSS in multivariable analysis. These findings raise the possibility that optic nerve head morphology may capture structural variation not fully reflected by conventional retinal thickness measures alone, although the biological basis of this association remains uncertain. Importantly, these associations remained consistent in sensitivity analyses restricted to EDSS–OCT intervals ≤180 days, suggesting that the observed findings were not solely driven by temporal mismatch between clinical and imaging assessments.

The observed inverse relationship between pRNFL thickness and CDR is consistent with the known structural consequences of axonal loss within the optic nerve. Retinal nerve fiber layer thinning reflects neuroaxonal degeneration, which is accompanied by neuroretinal rim loss and subsequent enlargement of the optic cup.

OCT-derived retinal parameters are well-established markers of neurodegeneration in MS, with several studies demonstrating their association with both physical and cognitive impairment [31]. In particular, early retinal thinning has been shown to predict disability progression in newly diagnosed MS patients [32]. Despite the structural association between pRNFL and CDR, pRNFL thickness was not associated with EDSS in this cohort. The lack of association between pRNFL thickness and disability may additionally reflect heterogeneity related to previous optic neuritis episodes, which could not be systematically controlled in this retrospective dataset. This is in line with previous studies reporting weak or inconsistent correlations between retinal thickness and global disability measures. One explanation is that EDSS predominantly reflects motor disability, whereas pRNFL captures localized neuroaxonal damage within the visual pathway. The present study was therefore intended as an exploratory assessment of whether retinal and optic nerve head structural measures may nevertheless reflect broader disease burden despite the known conceptual mismatch between visual pathway biomarkers and predominantly motor-oriented disability scales such as EDSS. This mismatch may partly explain why pRNFL thickness was not significantly associated with EDSS in the present cohort. Given the known heterogeneity of MS, structural damage in specific pathways may not directly translate into global disability scores [33]. Furthermore, OCT parameters have been shown to correlate more strongly with cognitive impairment than with EDSS [31]. Future studies incorporating MS Functional Composite (MSFC), low-contrast visual acuity testing, and cognitive metrics may provide a more sensitive assessment of the functional relevance of OCT-derived retinal biomarkers in MS.

An additional consideration is the apparent discordance between the lack of significant associations for pRNFL thickness and the observed associations between CDR and EDSS. Although optic cup enlargement is generally considered to accompany neuroretinal rim loss and axonal thinning, the inverse correlation observed between pRNFL thickness and CDR in the present cohort was weak (rho = −0.22). This suggests that factors other than retinal axonal loss may substantially contribute to CDR variability. In particular, physiological inter-individual differences in optic disc anatomy may influence CDR independently of MS-related pathology. Consequently, the present findings do not establish CDR as a specific marker of neurodegeneration in MS but rather support further investigation of CDR as an exploratory structural parameter that may reflect multiple biological and anatomical influences.

Importantly, the absence of systematically documented optic neuritis history represents a major limitation when interpreting both pRNFL and CDR findings. Both retinal nerve fiber layer thinning and optic nerve head remodeling may occur secondary to optic neuritis-related axonal loss, and detailed information regarding optic neuritis timing, severity, and laterality was not consistently available in this retrospective dataset. Consequently, the observed association between CDR and disability may partly reflect residual confounding related to prior optic neuritis rather than a direct relationship between optic nerve head morphology and global MS-related structural remodeling.

Although pRNFL thickness was not associated with EDSS in this cohort, CDR demonstrated consistent associations with both current disability and EDSS at follow-up. The use of area-based CDR captures both neuroretinal rim area and optic disc dimensions and may therefore reflect structural changes occurring at the level of the optic nerve head. Notably, baseline CDR remained independently associated with follow-up EDSS in multivariable analysis, although the biological basis of this association remains uncertain given the potential influence of optic neuritis history and physiological optic disc variability. In exploratory multivariable regression analyses, higher baseline CDR values remained associated with higher follow-up EDSS after adjustment for age, sex, and follow-up duration. However, given the non-normal residual distributions, these regression-derived effect estimates should be interpreted cautiously and considered supportive of the non-parametric correlation findings rather than confirmatory evidence. In addition, physiological variability in optic disc morphology may influence CDR measurements independently of neurodegenerative processes. Therefore, the present findings should not be interpreted as evidence that CDR represents a validated marker of MS-related neurodegeneration.

While CDR has traditionally been studied in glaucoma, its role in MS has received limited attention. CDR may reflect a combination of neuroaxonal loss, optic nerve head remodeling, and physiological inter-individual variability rather than a specific biomarker of MS-related neurodegeneration. However, the influence of optic disc size on CDR interpretation should be considered, as larger discs may physiologically exhibit higher CDR values independent of neurodegeneration. Consequently, the observed associations between CDR and disability may partly reflect anatomical variability and residual confounding rather than disease-specific neurodegenerative processes alone.

In addition to structural OCT parameters, we explored subjective visual vertical (SVV) as a functional measure of spatial orientation in MS. In the present study, the subtle misperception of verticality in the MS patient sample was observed exclusively under dynamic visual conditions. This finding is consistent with the known role of visual input in the estimation of gravitational vertical. Visual input plays an important role in spatial orientation and estimation of gravitational verticality [28,29]. Consistent with previous reports, dynamic visual stimulation increased SVV deviations in patients with MS, supporting the concept of altered multisensory integration in this population [23,30,34,35].

Importantly, no significant associations were identified between retinal structural measures and SVV performance under either static or dynamic conditions, nor with visual dependence scores. While this may suggest that retinal structural alterations are not the primary determinants of verticality perception abnormalities in MS, these findings should be interpreted cautiously. The SVV subgroup was relatively small (*n* = 37), and the analyses were exploratory and not powered to detect small-to-moderate associations. Therefore, the absence of significant correlations should not be interpreted as evidence that retinal structure is unrelated to verticality perception. Previous studies suggest that verticality perception depends on the integration of visual, vestibular, and somatosensory information within distributed central nervous system networks. Consequently, it is plausible that abnormalities in SVV may be influenced by disturbances of central sensory integration rather than retinal structural changes alone. However, the present data are insufficient to directly test this hypothesis. Future studies incorporating larger samples and complementary measures of visual, vestibular, and central sensory processing are needed to clarify the mechanisms underlying altered verticality perception in MS.

Neither pRNFL nor CDR changes were associated with changes in EDSS or annualized disability progression. This lack of association may reflect both biological and methodological factors, including the limited sensitivity of EDSS to detect short-term changes. From a biological perspective, structural changes detected by OCT are typically gradual and may not parallel short-term clinical fluctuations. From a methodological standpoint, EDSS has well-recognized limitations, including non-linearity and reduced sensitivity, particularly in early disease stages and in domains such as cognition and visual function [36].

Additionally, variability in OCT measurements and the relatively small longitudinal sample size may further limit the ability to detect subtle progression-related changes. The present findings suggest that CDR may provide clinically relevant information beyond traditional OCT parameters such as pRNFL. Given its association with both concurrent disability and EDSS at follow-up, CDR may warrant further investigation as an exploratory OCT-derived structural measure in MS. In our model, CDR, together with age, sex and follow-up duration, explained approximately 34% of the variance in EDSS (R^2^ = 0.34). These results align with growing evidence supporting the use of OCT as a non-invasive tool for monitoring neurodegeneration and disease progression [32]. Incorporating optic nerve head parameters such as CDR into routine OCT analysis may improve the clinical utility of this modality.

This study has several strengths, including a well-characterized cohort and the integration of structural (OCT), clinical (EDSS), and functional (SVV) measures. The main limitation of this study is the retrospective design and incomplete availability of detailed optic neuritis history, which limited the ability to account for optic neuritis-related effects on OCT parameters. The use of multivariable regression allowed adjustment for relevant confounders, strengthening the robustness of the findings. However, several limitations should be acknowledged. Not all patients had completed longitudinal OCT data, resulting in variable sample sizes across analyses. In addition, EDSS may not fully capture non-motor aspects of disability, particularly visual and cognitive impairment. Finally, the observational design precludes conclusions regarding causality. Because residual distributions deviated from normality, regression-based estimates should be interpreted cautiously and viewed primarily as exploratory analyses supporting the direction and magnitude of associations observed in the non-parametric correlation analyses. Additionally, OCT parameters were summarized at the patient level by averaging both eyes, which may reduce sensitivity to inter-eye differences, although sensitivity analyses suggested that this did not materially affect the results. Although sensitivity analyses restricted to EDSS–OCT intervals ≤ 180 days supported the robustness of the findings, temporal mismatch between clinical and OCT assessments remains a limitation of the retrospective design. Detailed information regarding optic neuritis timing, severity, and laterality was not consistently available in retrospective clinical records. Consequently, analyses could not be stratified according to optic neuritis history. Given the known effects of optic neuritis on OCT-derived retinal measures, findings should therefore be interpreted cautiously and considered exploratory. GCIPL measurements were not consistently available in the retrospective dataset and therefore could not be included in the present analyses. This represents an important limitation, as GCIPL has been reported to be a sensitive OCT marker of neuroaxonal injury and disability progression in MS and may have provided a valuable structural comparator for the observed CDR findings.

## 5. Conclusions

Higher CDR values, but not pRNFL thickness, were associated with disability measures in this exploratory retrospective MS cohort. However, CDR should be interpreted cautiously as an indirect and potentially confounded structural parameter, as optic neuritis history could not be systematically accounted for and physiological optic disc variability may substantially influence CDR measurements. SVV abnormalities were not associated with retinal structural measures in this exploratory subgroup analysis. Although verticality perception abnormalities in MS may be related to disturbances of central sensory integration, larger studies are needed to determine whether retinal structural changes contribute to these abnormalities and to further clarify the underlying mechanisms. Prospective longitudinal studies incorporating detailed optic neuritis characterization and additional retinal biomarkers are needed to validate these findings.

## Figures and Tables

**Table 1:** Clinical and OCT characteristics of the study.

Variable	Value
Demographic and clinical characteristics	
Age, years	34.6 ± 9.2 (*n* = 100)
EDSS at baseline	2.0 [1.5–2.5] (*n* = 100)
EDSS at follow-up	2.5 [2.0–3.5] (*n* = 100)
Annual EDSS change	0.10 [0.00–0.40] (*n* = 100)
Follow-up, years	2.9 [1.2–6.4] (*n* = 100)
OCT parameters	
pRNFL at baseline, µm	94.8 ± 14.1 (*n* = 100)
pRNFL at follow-up, µm	89.2 ± 15.5 (*n* = 57)
CDR at baseline	0.29 ± 0.19 (*n* = 83)
CDR at follow-up	0.31 ± 0.22 (*n* = 51)

Values are presented as mean ± standard deviation or median [interquartile range], as appropriate. Sample sizes vary due to missing data. EDSS—Expanded Disability Status Scale; OCT—Optical coherence tomography; pRNFL—peripapillary Retinal Nerve Fiber Layer; CDR—cup-to-disc ratio.

**Table 2:** Multivariable linear regression analysis of factors associated with follow-up EDSS.

Predictor	B (95% CI)	*p*-Value
CDR	2.59 (1.26–3.92)	0.0002
Age	0.04 (0.01–0.07)	0.005
Follow-up duration (years)	0.0002 (−0.00003–0.00038)	0.098
Sex	−0.23 (−0.77–0.32)	0.41

Values are presented as unstandardized regression coefficients (B) with 95% confidence intervals. Model R^2^ = 0.34; overall model *p* < 0.0001. EDSS—Expanded Disability Status Scale; CDR—cup-to-disc ratio.

**Table 3:** Correlations between structural retinal changes and verticality perception measures.

Variable	Static SVV	Dynamic CW	Dynamic CCW	VD
pRNFL thickness	0.021	0.177	0.075	−0.055
	*p* = 0.323	*p* = 0.274	*p* = 0.646	*p* = 0.738
CDR	0.018	−0.102	−0.113	−0.029
	*p* = 0.914	*p* = 0.55	*p* = 0.507	*p* = 0.863

Spearman correlation coefficients (rho) between structural retinal parameters and SVV measures in the SVV subgroup (*n* = 37). SVV—subjective visual vertical; CW—clockwise, CCW—counter-clockwise; VD—visual dependence; pRNFL—peripapillary retinal nerve fiber layer; CDR—cup to disk ratio.

## Data Availability

The data presented in this study are available on request from the corresponding author due to privacy and ethical reasons.

## Abbreviations

The following abbreviations are used in this manuscript:

CDR	Cup-to-Disc Ratio
OCT	Optical Coherence Tomography
MS	Multiple Sclerosis
EDSS	Expanded Disability Status Scale
SVV	Subjective Visual Vertical
CW	Clockwise
CCW	Counter-clockwise
VD	Visual Dependence

## Appendix A

**Table A1:** Worst-eye sensitivity analyses of OCT parameters and EDSS measures.

OCT Parameter (Worst Eye)	Outcome Measure	Spearman Rho	95% CI	*p*-Value	*n*
Baseline CDR	Baseline EDSS	0.32	[0.10–0.50]	0.0038	82
Baseline CDR	Follow-up EDSS	0.51	[0.32–0.66]	<0.0001	82
Baseline pRNFL	Baseline EDSS	−0.09	[−0.29–0.11]	0.359	98
Baseline pRNFL	Follow-up EDSS	−0.22	[−0.40–−0.02]	0.031	98

Abbreviations: CDR—cup-to-disc ratio; pRNFL—peripapillary retinal nerve fiber layer; EDSS—Expanded Disability Status Scale.

## References

[1] Villoslada P. (2010). Biomarkers for multiple sclerosis. Drug News Perspect..

[2] Gourraud P., Henry R.G., Cree B.A.C., Crane J.C., Lizee A., Olson M.P., Santaniello A.V., Datta E., Zhu A.H., Bevan C.J. (2014). Precision medicine in chronic disease management: The MS BioScreen. Ann. Neurol..

[3] Green A.J., McQuaid S., Hauser S.L., Allen I.V., Lyness R. (2010). Ocular pathology in multiple sclerosis: Retinal atrophy and in-flammation irrespective of disease duration. Brain.

[4] Britze J., Pihl-Jensen G., Frederiksen J.L. (2017). Retinal ganglion cell analysis in multiple sclerosis and optic neuritis: A systematic review and meta-analysis. J. Neurol..

[5] Petzold A., de Boer J.F., Schippling S., Vermersch P., Kardon R., Green A., A Calabresi P., Polman C. (2010). Optical coherence tomography in multiple sclerosis: A systematic review and meta-analysis. Lancet Neurol..

[6] Phuljhele S., Kedar S., Saxena R. (2021). Approach to optic neuritis: An update. Indian. J. Ophthalmol..

[7] Syc S., Warner C., Saidha S., Farrell S., Conger A., Bisker E., Wilson J., Frohman T., Frohman E., Balcer L. (2011). Cup to disc ratio by optical coherence tomography is abnormal in multiple sclerosis. J. Neurol. Sci..

[8] Gabilondo I., Martínez-Lapiscina E.H., Martínez-Heras E., Fraga-Pumar E., Llufriu S., Ortiz S., Bullich S., Sepulveda M., Falcon C., Berenguer J. (2014). Trans-synaptic axonal degeneration in the visual pathway in multiple sclerosis. Ann. Neurol..

[9] Balk L.J., Steenwijk M.D., Tewarie P., Daams M., Killestein J., Wattjes M.P., Vrenken H., Barkhof F., Polman C.H., Uitdehaag B.M.J. (2015). Bidirectional trans-synaptic axonal degenera-tion in the visual pathway in multiple sclerosis. J. Neurol. Neurosurg. Psychiatry.

[10] Filippatou A.G., Calabresi P.A., Saidha S., Murphy O.C. (2023). Spotlight on Trans-Synaptic Degeneration in the Visual Pathway in Multiple Sclerosis. Eye Brain.

[11] Abalo-Lojo J.M., Treus A., Arias M., Gómez-Ulla F., Gonzalez F. (2018). Longitudinal study of retinal nerve fiber layer thickness changes in a multiple sclerosis patients cohort: A long term 5 year follow-up. Mult. Scler. Relat. Disord..

[12] Schurz N., Sariaslani L., Altmann P., Leutmezer F., Mitsch C., Pemp B., Rommer P., Zrzavy T., Berger T., Bsteh G. (2021). Evaluation of Retinal Layer Thickness Parame-ters as Biomarkers in a Real-World Multiple Sclerosis Cohort. Eye Brain.

[13] Abalo-Lojo J.M., Limeres C.C., Gómez M.A., Baleato-González S., Cadarso-Suárez C., Capeáns-Tomé C., Gonzalez F. (2014). Retinal nerve fiber layer thickness, brain atrophy, and disability in multiple sclerosis patients. J. Neuroophthalmol..

[14] Ratchford J.N., Saidha S., Sotirchos E.S., Oh J.A., Seigo M.A., Eckstein C., Durbin M.K., Oakley J.D., Meyer S.A., Conger A. (2013). Active MS is associated with accelerated retinal ganglion cell/inner plexiform layer thinning. Neurology.

[15] Saidha S., Al-Louzi O., Ratchford J.N., Bhargava P., Oh J., Newsome S.D., Prince J.L., Pham D., Roy S., van Zijl P. (2015). Optical coherence tomography reflects brain atrophy in multiple sclerosis: A four-year study. Ann. Neurol..

[16] Kim H., Park J.E., Choi W. (2025). Establishment of Normative Retinal Nerve Fiber Layer Thickness in Healthy Koreans Using Huvitz Optical Coherence Tomography and Comparison with Cirrus OCT. J. Clin. Med..

[17] Cameron M.H., Nilsagard Y. (2018). Balance, gait, and falls in multiple sclerosis. Handb. Clin. Neurol..

[18] Marrie R.A., Cutter G.R., Tyry T. (2013). Substantial burden of dizziness in multiple sclerosis. Mult. Scler. Relat. Disord..

[19] Klatt B.N., Sparto P.J., Terhorst L., Winser S., Heyman R., Whitney S.L. (2019). Relationship between subjective visual vertical and balance in individuals with multiple sclerosis. Physiother. Res. Int..

[20] Cochrane G.D., Christy J.B., Motl R.W. (2021). Central Vestibular Functions Correlate With Fatigue and Walking Capacity in People With Multiple Sclerosis. Phys. Ther..

[21] da Fonseca B.A.V., Pereira C.B., Jorge F., Simm R., Apostolos-Pereira S., Callegaro D. (2016). A disturbed processing of graviceptive pathways may be involved in the pathophysiology of balance disorders in patients with multiple sclerosis. Arq. Neuro-Psiquiatr..

[22] Cochrane G.D., Christy J.B., Motl R.W. (2021). Comprehensive Clinical Assessment of Vestibular Function in Multiple Sclerosis. J. Neurol. Phys. Ther..

[23] Ulozienė I., Totilienė M., Balnytė R., Kuzminienė A., Kregždytė R., Paulauskas A., Blažauskas T., Marozas V., Uloza V., Kaski D. (2020). Subjective visual vertical and visual dependency in patients with multiple sclerosis. Mult. Scler. Relat. Disord..

[24] Oberwahrenbrock T., Schippling S., Ringelstein M., Kaufhold F., Zimmermann H., Keser N., Young K.L., Harmel J., Hartung H.-P., Martin R. (2012). Retinal Damage in Multiple Sclerosis Disease Subtypes Measured by High-Resolution Optical Coherence Tomography. Mult. Scler. Int..

[25] Schippling S., Balk L., Costello F., Albrecht P., Balcer L., Calabresi P., Frederiksen J., Frohman E., Green A., Klistorner A. (2015). Quality control for retinal OCT in multiple scle-rosis: Validation of the OSCAR-IB criteria. Mult. Scler. J..

[26] Klėgėris T., Kaski D., Balnytė R., Uloza V., Kuzminienė A., Ulozienė I. (2025). Toward Standardized Assessment of Dynamic Sub-jective Visual Vertical: Effects of Visual Stimulus Intensity in Health and Multiple Sclerosis. Medicina.

[27] Ulozienė I., Totilienė M., Paulauskas A., Blažauskas T., Marozas V., Kaski D., Ulozas V. (2017). Subjective visual vertical assessment with mobile virtual reality system. Medicina.

[28] Dakin C.J., Rosenberg A. (2018). Gravity estimation and verticality perception. Handb. Clin. Neurol..

[29] Nasr S., Tootell R.B.H. (2012). A cardinal orientation bias in scene-selective visual cortex. J. Neurosci..

[30] Dichgans J., Held R., Young L.R., Brandt T. (1972). Moving visual scenes influence the apparent direction of gravity. Science.

[31] Dreyer-Alster S., Gal A., Achiron A. (2021). Optical Coherence Tomography Is Associated With Cognitive Impairment in Multiple Sclerosis. J. Neuro-Ophthalmol..

[32] Toscano S., Chisari C.G., Biondi A., Patti F. (2024). Early reduction of retinal thickness predicts physical and cognitive disability in newly diagnosed multiple sclerosis patients: Results from a cross-sectional study. Neurol. Sci..

[33] Balk L.J., Cruz-Herranz A., Albrecht P., Arnow S., Gelfand J.M., Tewarie P., Killestein J., Uitdehaag B.M.J., Petzold A., Green A.J. (2016). Timing of retinal neuronal and axonal loss in MS: A longitudinal OCT study. J. Neurol..

[34] Dakin C.J., Peters A., Giunti P., Day B.L. (2018). Cerebellar Degeneration Increases Visual Influence on Dynamic Estimates of Verti-cality. Curr. Biol..

[35] Cousins S., Cutfield N.J., Kaski D., Palla A., Seemungal B.M., Golding J.F., Staab J.P., Bronstein A.M. (2014). Visual dependency and dizziness after vestib-ular neuritis. PLoS ONE.

[36] Sousa C., França M., Jacques T., José Sá M., Alves R.A. (2025). Longitudinal Study of Cognitive Phenotypes in Patients with Relaps-ing-Remitting Multiple Sclerosis. Arch. Clin. Neuropsychol..

